# Synergism Between IL21 and Anti-PD-1 Combination Therapy is Underpinned by the Coordinated Reprogramming of the Immune Cellular Network in the Tumor Microenvironment

**DOI:** 10.1158/2767-9764.CRC-23-0012

**Published:** 2023-08-04

**Authors:** Shaoxian Wu, Hao Huang, Runzi Sun, David Shihong Gao, Fan Ye, Jianing Huang, Ella Li, Andrew Ni, Kevin GuoKai Lu, Kong Chen, Jingting Jiang, Penelope A. Morel, Ziyang Zhong, Binfeng Lu

**Affiliations:** 1 Department of Immunology, University of Pittsburgh School of Medicine, Pittsburgh, Pennsylvania.; 2 Department of Tumor Biological Treatment, The Third Affiliated Hospital of Soochow University, Changzhou, P.R. China.; 3 Anwita Biosciences Inc, San Carlos, California.; 4 Pulmonary, Allergy and Critical Care Medicine, University of Pittsburgh School of Medicine, Pittsburgh, Pennsylvania.; 5 Center for Discovery and Innovation, Hackensack Meridian Health, Nutley, New Jersey.

## Abstract

T cell–stimulating cytokines and immune checkpoint inhibitors (ICI) are an ideal combination for increasing response rates of cancer immunotherapy. However, the results of clinical trials have not been satisfying. It is important to understand the mechanism of synergy between these two therapeutic modalities. Here, through integrated analysis of multiple single-cell RNA sequencing (scRNA-seq) datasets of human tumor-infiltrating immune cells, we demonstrate that IL21 is produced by tumor-associated T follicular helper cells and hyperactivated/exhausted CXCL13^+^CD4^+^ T cells in the human tumor microenvironment (TME). In the mouse model, the hyperactivated/exhausted CD4^+^ T cell–derived IL21 enhances the helper function of CD4^+^ T cells that boost CD8^+^ T cell–mediated immune responses during PD-1 blockade immunotherapy. In addition, we demonstrated that IL21’s antitumor activity did not require T-cell trafficking. Using scRNA-seq analysis of the whole tumor-infiltrating immune cells, we demonstrated that IL21 treatment in combination with anti-PD-1 blockade synergistically drives tumor antigen–specific CD8^+^ T cells to undergo clonal expansion and differentiate toward the hyperactive/exhausted functional state in the TME. In addition, IL21 treatment and anti-PD-1 blockade synergistically promote dendritic cell (DC) activation and maturation to mature DC as well as monocyte to type 1 macrophage (M1) differentiation in the TME. Furthermore, the combined treatment reprograms the immune cellular network by reshaping cell-cell communication in the TME. Our study establishes unique mechanisms of synergy between IL21 and PD-1–based ICI in the TME through the coordinated promotion of type 1 immune responses.

**Significance:**

This study reveals how cytokine and checkpoint inhibitor therapy can be combined to increase the efficacy of cancer immunotherapy.

## Introduction

T cells are the cardinal players in cancer immunotherapy. T cell–mediated immune responses are critically dependent on both antigen stimulation and cytokines. Cytokines as cancer immunotherapies, such as IL2 and IFNα, can activate and sustain T cell–mediated immunity against cancer cells (1). In contrast, the antitumor activities of immune checkpoint inhibitors (ICI) are dependent upon their ability to abrogate immune inhibitory signals on T cells (2). Thus, it seems intuitive that the coadministration of cytokines with ICIs should result in greater therapeutic effects. However, no benefits of any cytokine/ICI combination therapy have been observed in recent clinical trials (3, 4). There are many barriers to cytokine/ICI combination therapy, which include the dose-limiting toxicity of cytokines, antagonistic immune responses triggered by cytokines and ICIs, and differences in pharmacokinetics (1, 5–8). Therefore, the underlying mechanisms of synergism between cytokines and ICIs in cancer immunotherapy need to be better understood and maximized in future drug design.

This study provides insight into how systemically delivered IL21 exerts its antitumor functions alone and in combination with anti-PD-1 mAbs. To understand how IL21 is involved in cancer immunity in humans and mice, we first investigated the cellular origin of IL21 in human cancer tissues and mouse syngeneic tumor models by integrating and analyzing multiple single-cell RNA sequencing (scRNA-seq) datasets. Then, we determined whether IL21 is required for anti-PD-1 therapy in a mouse model. In addition, we investigated whether the antitumor function of IL21 and PD-1 blockade depends primarily on immune responses in the tumor or peripheral lymphoid organs. Furthermore, we utilized scRNA-seq and paired single-cell T-cell receptor sequencing (scTCR-seq) to study synergism between IL21 and PD-1 blockade in the tumor microenvironment (TME).

## Materials and Methods

### Mouse

The 6–8 weeks old female C57BL/6 mice used in experiments were purchased from The Jackson Laboratory and were bred and raised in the Animal Center of the University of Pittsburgh School of Medicine at the specific pathogen-free level. All mouse experiments in this study have been reviewed and approved by the Animal Care Committee of the University of Pittsburgh (Pittsburgh, PA).

### Cell Culture and Tumor Model

MC38 (RRID:CVCL_B288) cell line was generously provided by Dr. Zongsheng Guo (University of Pittsburgh, School of Medicine, Pittsburgh, PA), were cultured in a complete DMEM containing 1% penicillin-streptomycin and 10% FBS. All cells were cultured in a constant temperature incubator at 37°C with 5% CO_2_, and the supernatant of tumor cells will be collected regularly for *Mycoplasma* detection. MC38 cells undergo passage every 2 days, and their growth characteristics and morphology are assessed by well-trained senior laboratory personnel. The passage history of the cells is carefully maintained, and only cells with a passage number below 10 are utilized for experiments. To facilitate comprehensive tracking, a centralized cell bank database is used to maintain a digital record of all cell lines in the laboratory. MC38 has not been authenticated by outside genetic services. MC38 cells in the best growth state were prepared such that a 50 μL cell suspension (1 × 10^6^ cells/mouse) was inoculated subcutaneously in the abdomen of 6–8 weeks old female C57BL/6 mice. When the subcutaneously transplanted tumor volume reached about 60 mm^3^, mice were randomly divided into control and treatment groups, namely, IgG, IL21-anti-HSA (anti-human serum albumin), anti-PD-1, IL21-anti-HSA/anti-PD-1, intraperitoneal administration was performed, and the dosage of IL21-anti-HSA was 25 μg per mouse and anti-PD-1 200 μg per mouse, once every 4 days for a total of four times. The mice were also randomly divided into the control group and the treatment group, and the mice were given intraperitoneal administration of FTY720, IL21-anti-HSA, FTY720/IL21-anti-HSA, or DMSO. FTY720 was administered to each mouse according to its body weight at 1 mg/kg. Each mouse was treated with 25 μg of IL21-anti-HSA every 4 days for a total of four times. The mice were also randomly divided into control group and treatment group, and administered intraperitoneally with anti-PD-1 mAb, IL21R mAb, anti-PD-1 mAb/anti-IL21R mAb, and IgG isotype control. A total of 200 μg of anti-PD-1 mAb or isotype control per mouse was used. Each mouse was treated with 25 μg of IL21-anti-HSA every 4 days for a total of four times. The long and short diameters of tumors were measured every 2 days to generate tumor growth curves for the mice. The mouse tumor size was calculated as follows: *L* × *S*^2^/2 (*L* is the long diameter, *S* is the short diameter).

### Reagents and Antibodies

#### *In Vivo* Studies

IL21-anti-HSA, half-life–extended-IL21, was kindly provided by Anwita Biosciences. Anti-IL21R mAb (catalog no. BE0258, RRID:AB_2687737), anti-PD-1 mAb (catalog no. BE0033-2, RRID:AB_1107747), and hamster IgG (catalog no. BE0091, RRID:AB_1107773) were purchased from BioxCell. Flow cytometry: anti-CD45 (catalog no. 564279, RRID:AB_2651134), anti-CD4 (catalog no. 612844, RRID:AB_2870166), anti-CD8 (catalog no. 100722, RRID:AB_312761), anti-KI67 (catalog no. 100722, RRID:AB_312761), anti-IL21R (catalog no. 131906, RRID:AB_1279430) antibodies were purchased from BioLegend and BD Biosciences. Zombie NIR dye (catalog no. 423106) was purchased from BioLegend. Ghost Dye Violet 510 was purchased from Cell Signaling Technology (catalog no. 59863S). FTY720 (CAS# 402615-91-2) was purchased from Cayman Chemical.

### Processing of Tissues and Flow Cytometry

For mouse tumor processing and staining steps, please refer to our previous article (8). The mice were sacrificed, and tumors were excised, placed in 6-well plates containing 1.5 mL of serum-free RPMI1640, and cut into small pieces. Digestion was performed with 0.25 mg/mL Liberase TL (Roche) and 0.33 mg/mL DNase 1 (Sigma) at 37°C for 30 minutes, then terminated with complete medium (10% FBS RPMI1640). The single-cell suspension was filtered through a 40 μm cell strainer, washed with PBS, resuspended in 1% FBS HBSS, and added to a 96-well plate for staining. Flow cytometry analysis was performed with an Aurora (Cytek Biosciences) and analyzed using Flowjo software (FlowJo, RRID:SCR_008520). Tumor-infiltrating CD45^+^ immune cells were sorted by FACSAria II (BD Biosciences).

### Gene Expression and TCR Profiling by 5′ scRNA-seq

Single-cell suspensions were isolated from the MC38 tumor tissues, viability stained in PBS and surface stained for sorting in FACS buffer. In the surface stain of each individual sample, combinations of four cell hashing antibodies (BioLegend, TotalSeq-C0001-0004) were spiked in to label each individual sample. Cells were incubated for 30 minutes on ice and washed twice before sorting on live, TCRb^+^, CD45^+^. All tumor-infiltrating CD45^+^ T cells were sorted into one 15-mL conical tube with complete RPMI, and resuspended in 0.04% BSA, counted and loaded into a Single Cell Chip and processed through the 10x controller for droplet generation and Library preparation.

### 10x Genomics Library Preparation for 5′ scRNA-seq and TCR-seq

10x genomics 5′ Single Cell V(D)J + 5′ Gene Expression + Cell hashing libraries were generated as described in the User's Guide for 10x Chromium Single Cell V(D)J Reagent Kits. In brief, cells were subjected to in-drop lysis and reverse transcription, generating cDNA derived from mRNA in each cell and from the oligonucleotide-tagged cell hashing antibody, bearing bead-specific sequences to identify the cell of origin. cDNA was then amplified, and solid phase reversible immobilization (SPRI) selection was performed for downstream library construction for gene expression, TCR-seq and cell hashing library generation. Gene expression, TCR, and cell hashing libraries were pooled and sequenced using the NextSeq 500, the sequencing was performed at the University of Pittsburgh Genomics Center.

### Single-cell Transcriptome Sequencing Data Processing

The FASTQ files obtained by single-cell transcriptome sequencing were quantitatively analyzed using Cellranger software (version: 4.0) and the mm10 mouse reference genome. Cells were subjected to further quality control based on total unique molecular identifiers (UMI) count, number of genes detected and proportion of mitochondrial gene counts per cell, and proportion of ribosomal gene counts per cell (removal of UMI ≥ 30,000, and mitochondrial gene counts ≥10%, and cells with a ribosomal gene counts ≥50%).

The obtained filtered cells were subjected to principal component analysis, uniform manifold approximation projection (UMAP) dimensionality reduction, and unsupervised clustering analysis according to the official Seurat process, and the dimensionality-reduced population was identified according to the known characteristic genes of the mouse immune cell population. All visualization results are analyzed using the visualization functions in R package Seurat and Python package Scanpy (IPython, RRID:SCR_001658).

### Analysis and Visualization of Differentially Expressed Genes

Differential gene expression between different samples was analyzed using the edgeR package. The original count matrix obtained from the Seurat object was normalized using TMM (trimmed mean of M values) by the calcNormFactors function, and the dispersion of gene expression values was estimated using the estimateDisp function. Then, the selected differentially expressed genes (DEG) were visualized using the Dotplot function or pheatmap package.

### Trajectory Analysis of T and Myeloid Cells

The monocle3 package was used to convert the Seurat object of CD8^+^ T cells and myeloid cells into the corresponding cds objects, and the developmental trajectory was constructed through the learn_graph function. The plot_cells function was used to visualize the analysis results.

### Analysis of scTCR-seq Data and Calculation of TCR Clonal Expansion

The FASTQ files obtained by single-cell transcriptome sequencing were quantitatively analyzed using the Cellranger vdj function and the mouse vdj reference sequence, and the generated filtered files were used for downstream analysis. Clonal expansion was defined by which clone size of TCR clonotype was greater or equal to 2. Expanded average was calculated by ratio of expanded TCR size and expanded clonotypes. The statistical results were calculated using the ggplot2 package (ggplot2, RRID:SCR_014601).

### Statistical Analysis

Graphing and statistical analysis were performed using GraphPad Prism 8.0 software (GraphPad Prism, RRID:SCR_002798). Data were expressed as mean ± SEM. According to different experimental conditions, one-way ANOVA was used for quantitative data comparison among multiple groups. Tumor growth curves between different groups were compared using two-way ANOVA. Data were considered statistically significant when the *P* value was less than 0.05. Values of *P* < 0.05 were ranked as *, *P* < 0.05; **, *P* < 0.01; ***, *P* < 0.001.

### Data Availability

Raw data for all analyzes presented in this study are available from the corresponding authors upon reasonable request.

### Ethics Statement

The animal study was reviewed and approved by the Institution Animal Care and Use Committee at University of Pittsburgh (Pittsburgh, PA).

## Results

### IL21 is Produced by Both CXCR5^+^ T_fh_ Cells and Hyperactivated/Exhausted CXCL13^+^CD4^+^ T Cells in the Human TME

**FIGURE 1:**
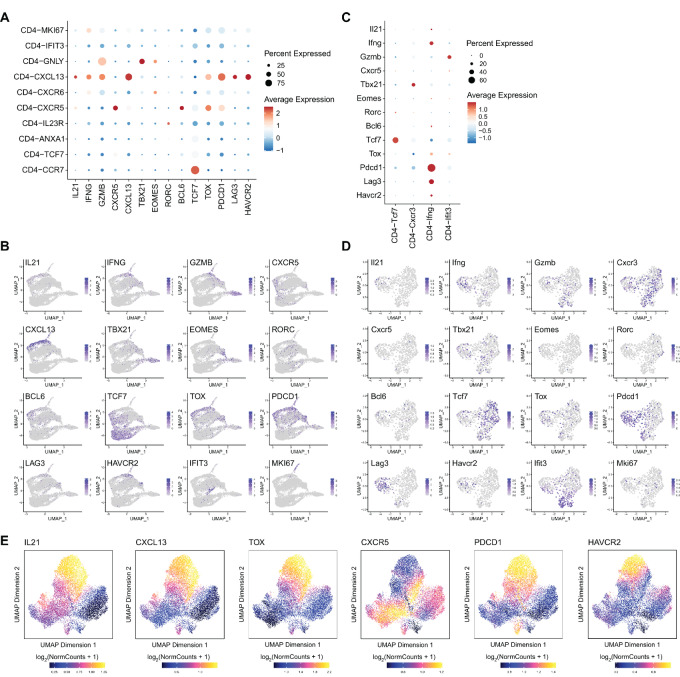
IL21 is produced by both CXCR5^+^ T_fh_ cells and hyperactivated/exhausted CD4^+^ T cells in the TME. **A** and **B,** Dot plots and UMAPs showing the expression of selected genes in different CD4^+^ T-cell subsets in integrated human cancer tissues. **C** and **D,** Dot plots and UMAPs showing the expression of selected genes in different CD4^+^ T-cell subsets in mouse MC38 colon adenocarcinoma tumors. **E,** UMAP plot showing imputed expression of selected genes constructed by ArchR.

To determine the cellular origin of IL21 in human tumor tissues, we analyzed public scRNA-seq data of 8,530 T cells from 12 patients with colon cancer, 9,055 T cells from 14 patients with non–small cell lung cancer, and 5,063 T cells from 6 patients with liver cancer (9–11). The analysis showed that IL21 is mainly expressed in two CD4^+^ T-cell subsets: CXCR5^+^CD4^+^ T cells and CXCL13^+^CD4^+^ T cells (Fig. 1A; Supplementary Fig. S1A). Expression of IL21 in CXCR5^+^CD4^+^ T cells is consistent with the notion that IL21 is highly expressed in T follicular helper (T_fh_) cells (12). CXCL13^+^CD4^+^ T cells highly express multiple immune checkpoint molecules, such as *PDCD1*, *LAG3*, *HAVCR2*, and cytotoxic effector molecules, such as *IFNG* and *GZMB* (Fig. 1A and B), indicating these cells are hyperactivated or exhausted (13–17). To determine the IL21-producing cells in mouse tumors, we analyzed public scRNA-seq data of CD4^+^ T cells in the MC38 colon adenocarcinoma model (18). The results showed that IL21 was predominantly expressed in a CD4^+^ T-cell subset that highly expressed *Pdcd1*, *Lag3*, and *Ifng*, consistent with a phenotype of hyperactivated or exhausted T cells (Fig. 1C and D; Supplementary Fig. S1B). It is worth noting that there was almost no T_fh_ subset in the TME of MC38 tumors (Fig. 1C and D). Collectively, we demonstrated that, besides T_fh_ cells, IL21 is expressed in hyperactivated/exhausted CD4^+^ T cells in many types of tumors.

We also analyzed single-cell Assay for Transposase-Accessible Chromatin using sequencing (scATAC-seq) data of human basal cell carcinoma (BCC; ref. 19). We found that the IL21 locus is accessible in both T_fh_ cells and hyperactivated/exhausted CD4^+^ T cells (Fig. 1E). These data further substantiated the conclusion that IL21 can be expressed by both T_fh_ cells and hyperactivated/exhausted CD4^+^ T cells.

### IL21 is Induced by Anti-PD-1 mAb and is Required for the Antitumor Efficacy of PD-1 Blockade Immunotherapy

**FIGURE 2:**
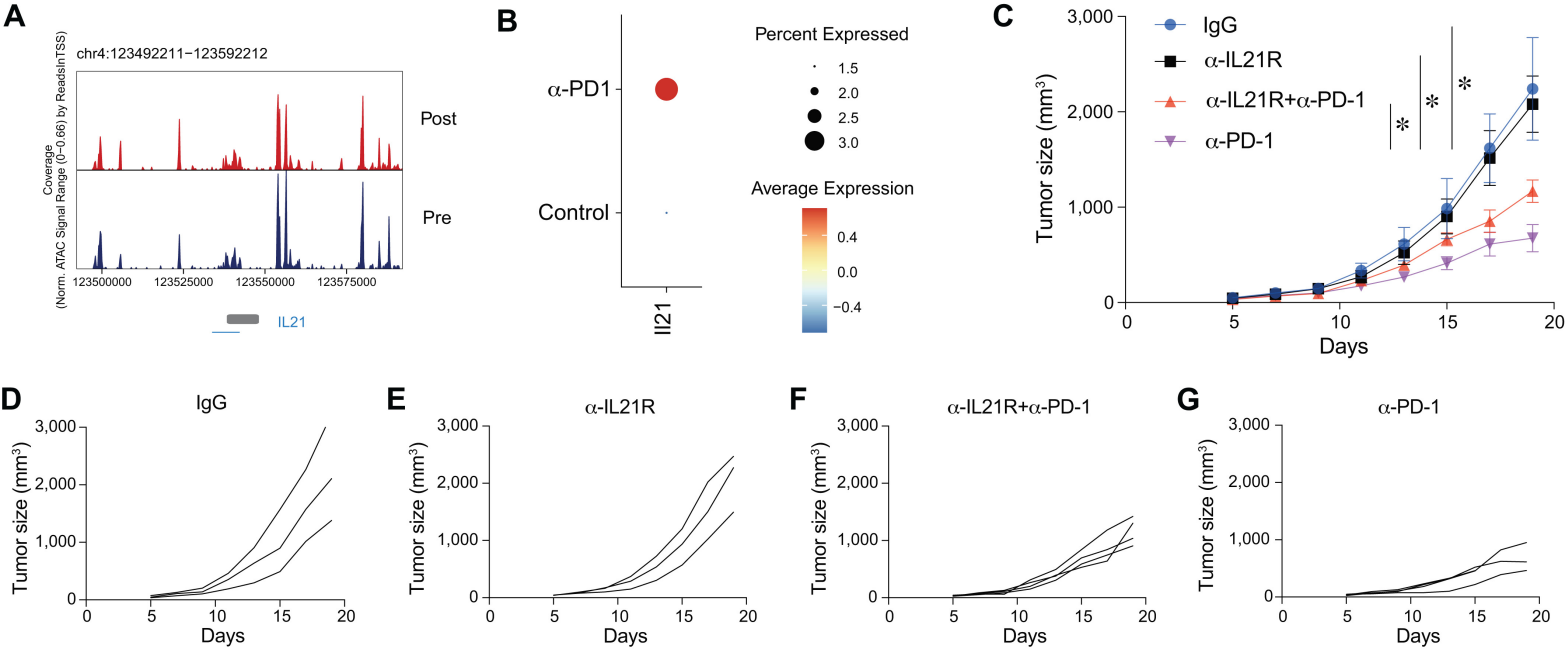
IL21 is induced by anti-PD-1 mAb and is required for the antitumor efficacy of PD-1 blockade immunotherapy. **A,** Genome tracks of aggregate scATAC-seq data at the *Il21* locus, clustered by different treatment conditions. **B,** Dot plot showing *Il21* expression in CD4^+^ T cells after PD-1 mAb treatment. **C,** Randomization of mice 5 days after inoculation with MC38 tumors, followed by intraperitoneal injection of drugs. Injections were given every 4 days for a total of four injections. Mouse tumor growth curves are shown in the figure (*n* = 4–5). **D**–**G,** Tumor growth curves of single mice in different groups. Data are presented as mean ± SEM, and two-way ANOVA test was used to compare the statistical differences of tumor growth curves among multiple groups. *, *P* < 0.05; **, *P* < 0.01; ***, *P* < 0.001; ****, *P* < 0.0001.

Because IL21 is expressed on CD4^+^ T cells along with multiple checkpoint molecules, we were interested in determining whether PD-1 blockade can increase its expression. Analysis of the human BCC scATAC-seq data revealed that accessibility at the IL21 locus increased upon anti-PD-1 mAb treatment (Fig. 2A). In addition, in the mouse MC38 scRNA-seq data, it was found that indeed the expression of IL21 in CD4^+^ accessibility T cells increased after anti-PD-1 mAb treatment (Fig. 2B). IL21 is expressed by exhausted CD4^+^ T cells (Supplementary Fig. S1C–S1E). We next explored whether endogenous IL21 is required for anti-PD-1 mAb-mediated antitumor responses. The results showed that administration of anti-IL21R mAb inhibited the antitumor effect of anti-PD-1 mAb treatment (Fig. 2C). These results showed that anti-PD-1 increased IL21 expression in CD4^+^ T cells, and blocking IL21R attenuated the antitumor effect of anti-PD-1 mAb (Fig. 2C–G). These data demonstrated that IL21 is involved in mediating the antitumor efficacy of anti-PD-1 mAb.

### Systemic Injection of IL21-anti-HSA Promoted Tumor site–focused Antitumor Activities

**FIGURE 3:**
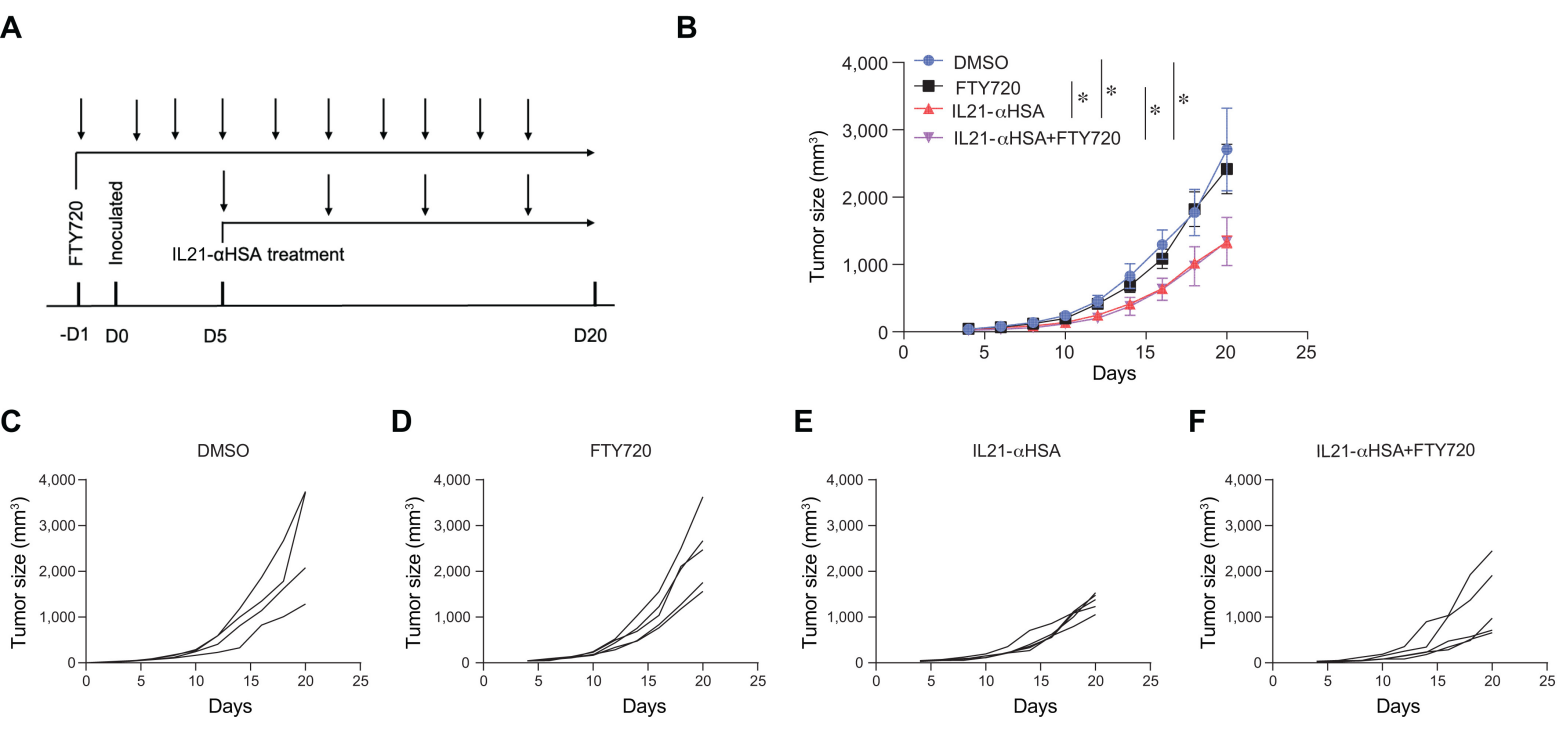
Systemic injection of IL21-anti-HSA promoted tumor site–focused antitumor activities. **A,** Diagram of the experimental protocol. C57BL/6 mice were injected intraperitoneally with FTY720 the day before tumor inoculation, and injected every other day. The next day, mice were inoculated with MC38 colon adenocarcinoma cells subcutaneously, and 5 days later, IL21-anti-HSA was injected intraperitoneally. Treatments were given every 4 days for a total of four treatments. Mouse tumor growth curves (*n* = 4–5) were drawn. **B,** Mouse tumor growth curve. **C**–**F,** The tumor growth curve of a single mouse in the above different treatment groups. Data are presented as mean ± SEM, and two-way ANOVA test was used to compare statistical differences in tumor growth curves between different groups. *, *P* < 0.05; **, *P* < 0.01; ***, *P* < 0.001; ****, *P* < 0.0001.

Systemic delivery of IL21 inhibits tumor growth by increasing antitumor immune responses (20–23). It is thought that T cells are activated in the draining lymph nodes and then migrate to the TME to exert their antitumor effector functions. Administration of IL21 by an intraperitoneal or intravenous route can reach both secondary lymphoid organs as well as the tumor. To resolve the sites where IL21 exerts its antitumor functions, we injected mice with FTY720, a S1P receptor inhibitor that blocks T-cell trafficking (24). We found that FTY720 injection did not affect the antitumor activity of IL21-anti-HSA, a pharmacokinetically improved IL21 (ref. 8; Fig. 3A–F; Supplementary Fig. S2A). On this basis, we also found that FTY720 injection did not affect anti-PD-1 and combination therapy antitumor effect (Supplementary Fig. S2B and S2D). These results suggest that T-cell trafficking is not required for IL21’s antitumor function, indicating that IL21 exerts its antitumor effect directly and primarily on the immune cells in the TME.

IL21-anti-HSA/anti-PD-1 mAb combination therapy increased cell-cycle genes, enhanced CD8^+^ T-cell clonal expansion, and promoted hyperactivated/exhausted T cells that express multiple checkpoint molecules.

We and others have shown that IL21 administration can be combined with PD-1 blockade to synergistically inhibit tumor growth (8, 25, 26). To gain a comprehensive understanding of how IL21 and PD-1 blockade together influence the immune network in the tumor, we performed scRNA-seq of tumor-infiltrating CD45^+^ immune cells sorted from MC38 tumors in mice treated with control IgG mAb, anti-PD-1 mAb, IL21-anti-HSA, and anti-PD-1 mAb/IL21-anti-HSA. We obtained 8,021 CD45^+^ immune cells in total, which allowed us to successfully subdivide these cells into T, B, myeloid, and natural killer (NK) cells (Supplementary Fig. S3A–S3E).

**FIGURE 4:**
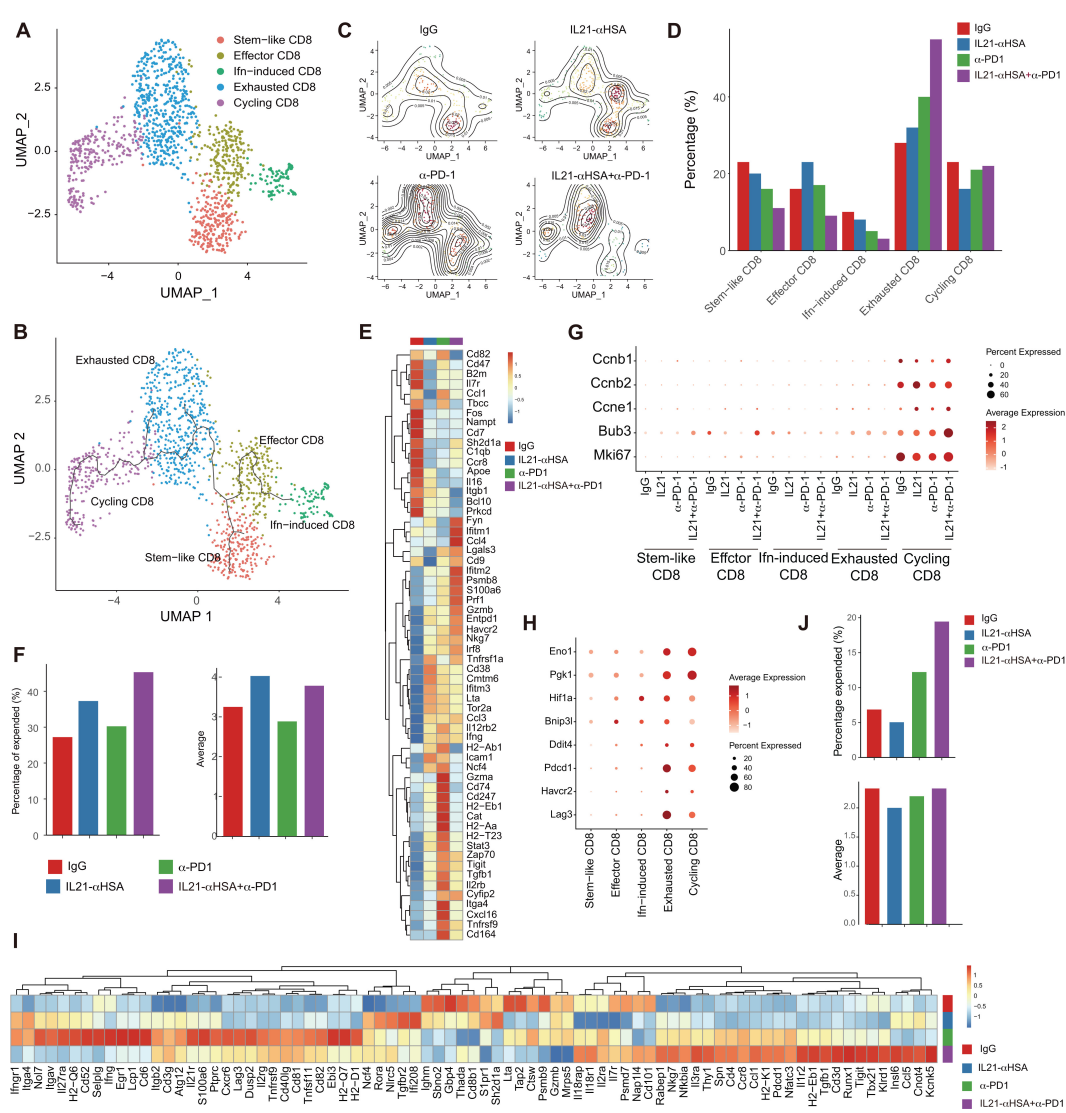
IL21-anti-HSA/PD-1 mAbs combination therapy alters T-cell function and increases T-cell clonal expansion. **A,** UMAP showing CD8^+^ T-cell subsets, including exhausted, cycling, stem-like, effector, and IFN-induced. **B,** Pseudotime plot of CD8^+^ T-cell subsets constructed by Monocle3. **C,** Contour heat map of CD8^+^ T-cell subsets split by different treatment conditions. **D,** Bar plots of CD8^+^ T-cell subsets under different treatment conditions. **E,** Heat map showing selected DEGs on exhausted CD8^+^ T cells in different treatment conditions. **F,** Bar plots showing TCR clonal expansion fractions and average clonal sizes of total CD8^+^ T cells under different treatment conditions. **G,** Dot plot showing expression of selected genes associated with the cell cycle on different CD8^+^ T-cell subsets under different treatment conditions. **H,** Dot plot showing expression of selected genes associated with glycolysis on different CD8^+^ T-cell subsets. **I,** Heat map showing expression of selected DEGs in CD4^+^ T cells under different treatment conditions. **J,** TCR clonal expansion fractions and average clonal sizes of CD4^+^ T cells under different treatment conditions.

We further analyzed the scRNA-seq data to determine how IL21 in combination with PD-1 blockade together affect tumor-infiltrating CD8^+^ T cells. Clustering analysis revealed five CD8^+^ T-cell subsets, including stem-like, IFN-induced, effector, exhausted, and cycling (Fig. 4A). Trajectory analysis suggested that CD8^+^ T cells followed a differentiation route from stem-like to effector and ultimately to exhausted and cycling cells. Another differentiation route consisted of stem-like and IFN-induced CD8^+^ T cells (Fig. 4B). IL21-anti-HSA and anti-PD-1 mAb impacted the first developmental route and synergistically decreased the fractions of stem-like and effector CD8^+^ T cells but increased the frequencies of exhausted CD8^+^ T cells (Fig. 4C and D). In addition, combined treatment also resulted in a decrease in the fraction of IFN-induced CD8^+^ T cells (Fig. 4C and D). We further analyzed DEGs in the exhausted subset. IL21-anti-HSA and anti-PD-1 mAb synergistically induced a decrease in multiple genes associated with a resting phenotype, such as *Il7r* and *Tcf7* (Fig. 4E). Moreover, IL21-anti-HSA administration and anti-PD-1 mAb synergistically induced expression of cytotoxic molecules such as *Prf1* and *Gzmb*, chemokines such as *Ccl3* and *Ccl4*, as well as checkpoint molecules such as *Entpd1* and *Havcr2* (Fig. 4E). These data suggest that IL21-anti-HSA and anti-PD-1 mAb drive an increased hyperactivated functional state that is controlled by multiple checkpoint molecules. In addition, an increase in *Ccl3* and *Ccl4* can also lead to greater interaction between CD8^+^ T cells and other immune cells in the TME.

The paired scTCR-seq also allowed analysis of CD8^+^ T-cell clonal expansion in these conditions. IL21-anti-HSA increased both the fraction of clonally expanded CD8^+^ T cells as well as the average clonal size of the expanded cells. Anti-PD-1 mAb alone modestly increased clonal expansion, but IL21-anti-HSA plus anti-PD-1 mAb further increased clonal expansion (Fig. 4F). We further examined cell cycle–related genes in the various CD8^+^ T-cell subsets and found that IL21-anti-HSA and IL21-anti-HSA in combination with anti-PD-1 mAb increased several cell cycle–related genes in the cycling cluster (Fig. 4G). We further studied how IL21-anti-HSA and anti-PD-1 mAb treatment affected proliferation of CD8^+^ T cells in the TME by flow cytometry. Our data showed that IL21-anti-HSA increased proliferation of IL21R^+^ CD8^+^ T cells (Supplementary Fig. S4A–S4C). Interestingly, anti-PD-1 mAb induced IL21R on CD8^+^ T cells and combined IL21-anti-HSA plus anti-PD-1 mAb treatment led to a further increase of proliferation particularly in IL21R^+^CD8^+^ T cells (Supplementary Fig. S4A and S4D). These data suggest that the observed synergy between IL21 and anti-PD-1 mAb in proliferation may be due to the upregulation of IL21R by anti-PD-1 mAb treatment and that IL21 directly promotes the proliferation of IL21R^+^ CD8^+^ T cells.

Our data indicated that proliferative CD8^+^ T cells were similar to exhausted cells in terms of expression of exhaustion markers and hypoxia signatures, suggesting their infiltration to hypoxic niches. However, these cells differ from exhausted cells by expressing proliferation genes, such as cyclins, *Bub3*, and *Mki67* (Fig. 4G and H). Our data showed that cycling genes were upregulated after combined IL21-anti-HSA and anti-PD-1 mAb treatment, suggesting that IL21-anti-HSA and anti-PD-1 mAb in combination can promote the cell cycle in hyperactivated/exhausted CD8^+^ T cells. This is consistent with our data that clonal expansion and increased fraction of exhausted T cells were greater in the combination group.

### IL21-anti-HSA and Anti-PD-1 mAb Synergistically Increased Cytotoxic CD4^+^ T Cells and CD4^+^ T-cell Clonal Expansion in the TME

We also analyzed the scRNA-seq data to determine how IL21, in combination with PD-1 blockade together, affects tumor-infiltrating CD4^+^ T cells in the TME. The scRNA-seq analysis showed that IL21-anti-HSA and anti-PD-1 mAb synergically increased a unique list of genes containing transcription factors *Tbx21*, *Nfatc3*, and *Nfkbia*, signaling molecules such as *Cd3d*, *Il2ra*, *Il18r1*, and *IL18rap*, cytolytic effector molecules such as *Gzma*, *Prf1*, and *Nkg7*, chemokines such as *Ccl5* and *Ccl1,* and checkpoint molecules such as *Pdcd1* and *Tigit*. These genes are likely involved in mediating the cytolytic functions of CD4^+^ T cells. In addition, checkpoint molecules, such as *Pdcd1* and *Tigit*, were also synergistically induced by IL21-anti-HSA and anti-PD-1 mAb. In contrast, *S1pr1* was diminished synergistically by IL21-anti-HSA and anti-PD-1 mAb coadministration, suggesting an increased local retention of CD4^+^ T cells (Fig. 4I). Besides these transcriptional changes, the percentage of clonally expanded CD4^+^ T cells was also increased by anti-PD-1 mAb and further enhanced by the combination of IL21-anti-HSA and anti-PD-1 mAb (Fig. 4J). Collectively, these data demonstrate that IL21-anti-HSA and anti-PD-1 mAb synergistically enhance a tumor site–focused CD4^+^ T cell–mediated antitumor immune response through promoting the hyperactivated/exhausted CD4^+^ T-cell subtype and CD4^+^ T-cell clonal expansion.

### IL21-anti-HSA and Anti-PD-1 mAb Administration Drives Phenotypic Polarization and Clonal Expansion of Regulatory T Cells

**FIGURE 5:**
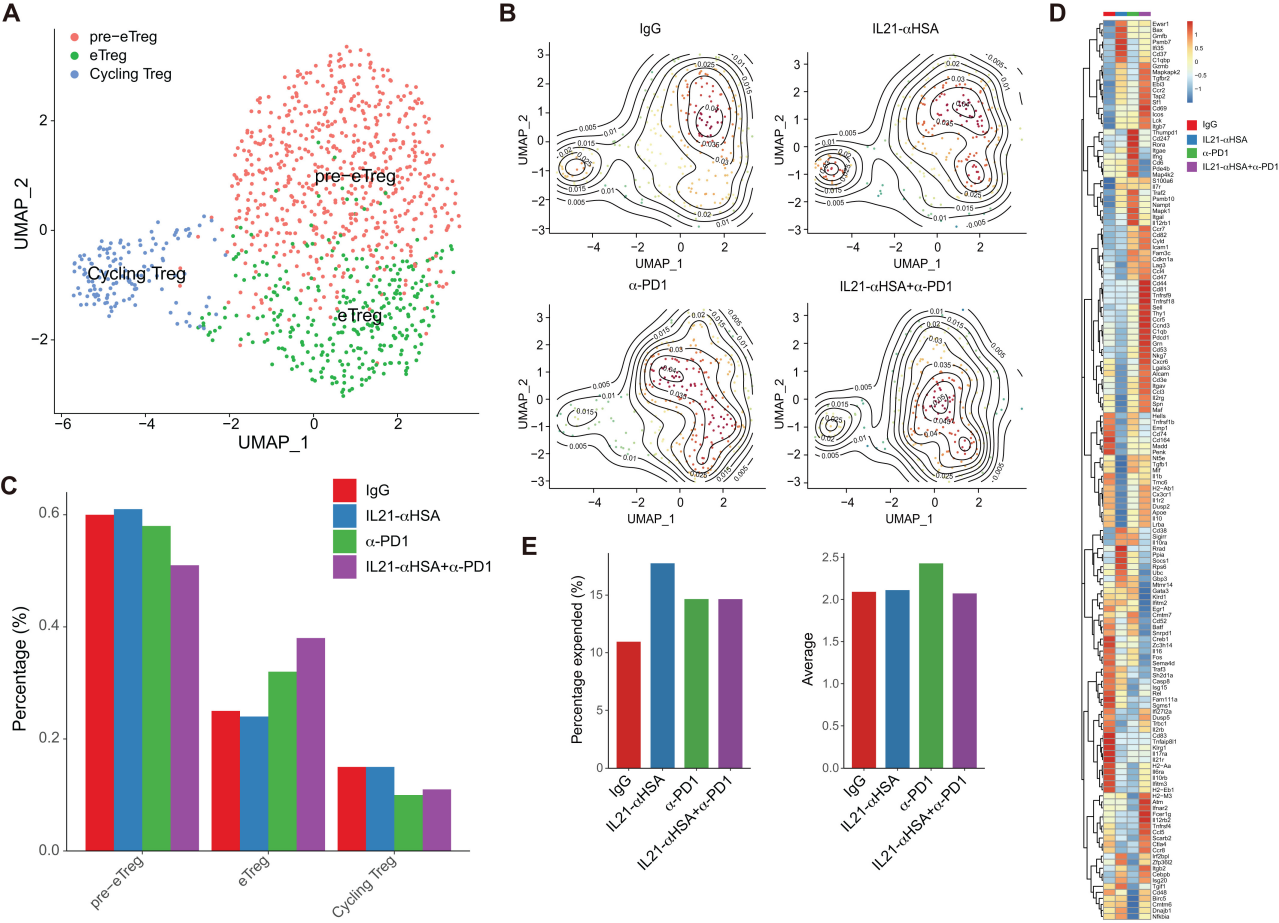
IL21-anti-HSA and anti-PD-1 mAb administration drives phenotypic polarization and clonal expansion of Treg cells. **A,** UMAP showing Treg cell subsets, including pre-eTreg cells, eTreg cells, and cycling Treg cells. **B,** Contour heat map of Treg cell subsets split by different treatment conditions. **C,** Bar plot of Treg cell subsets under different treatment conditions. **D,** Heat map showing expression of selected DEGs in Treg cells. **E,** TCR clonal expansion fractions and average clonal sizes of total Treg cells under different treatment conditions.

We then analyzed the scRNA-seq data to determine how IL21 in combination with PD-1 blockade together affect tumor-infiltrating regulatory T (Treg) cells. We firstly divided tumor-infiltrating Treg cells into three subpopulations according to characteristic genes and showed the distribution density of cell subpopulations among different treatment groups: pre-effector Treg (pre-eTreg) cells, effector Treg (eTreg) cells, and cycling Treg cells (Fig. 5A and B). After combined treatment, the proportion of pre-eTreg cells decreased, the proportion of eTreg cells increased, and the proportion of cycling Treg cells did not change much (Fig. 5C).

Consistent with this finding, differential gene expression analysis revealed that there are two clusters of genes that showed the greatest increase with combination treatment and either no increase or slight increase with IL21-anti-HSA and anti-PD-1 mAb treatment alone. The first cluster contains genes that are increased by IL21-anti-HSA and anti-PD-1 mAb treatment and further increased by combination treatment. These include transcription factors such as *Cebpb*, signaling molecules such as *Lck*, cytokine receptors such as *Il7r*, *Ccr2*, *Tgfbr2*, and *Ifnar2*, costimulatory molecules such as *Icos* and *Cd69*, and Treg cell effector molecules such as *Ebi3* and *Gzmb*. Another group of genes are mostly affected by anti-PD-1 mAb treatment, including *Pdcd1*, *Havcr*2, *Lag3*, *Il2rg*, *Cd44*, *Cd47*, *Ccr5*, *Tnfrsf18*, *Tnfrsf9* (also called 4-1BB), *Ccl3*, and *Ccl4* (Fig. 5D). Interestingly, IL21-anti-HSA treatment led to a decrease of several Treg effector molecules, such as *Ctla4*, *Il10*, *Tgfb1*, *Il1r2*, and *Penk*, but an increase of *Il1rl1*, indicating that IL21-anti-HSA also regulated the tumoral abundance of different Treg cell subtypes (Fig. 5D). In addition, TCR analysis revealed that both IL21-anti-HSA and anti-PD-1 mAb led to an increase in clonal expansion of Treg cells (Fig. 5E). Collectively, these results suggest that IL21-anti-HSA and anti-PD-1 mAb impact the differentiation of Treg cells by increasing Treg cells producing *Ebi3*, *Lag3*, and *Gzmb* but reducing Treg cells producing effector molecules, such as *Il10, Tgfb1, Ctla4,* and *Il1r2*, and enhancing clonal expansion.

### Combination Therapy Enhances Dendritic Cell Activation and Maturation

**FIGURE 6:**
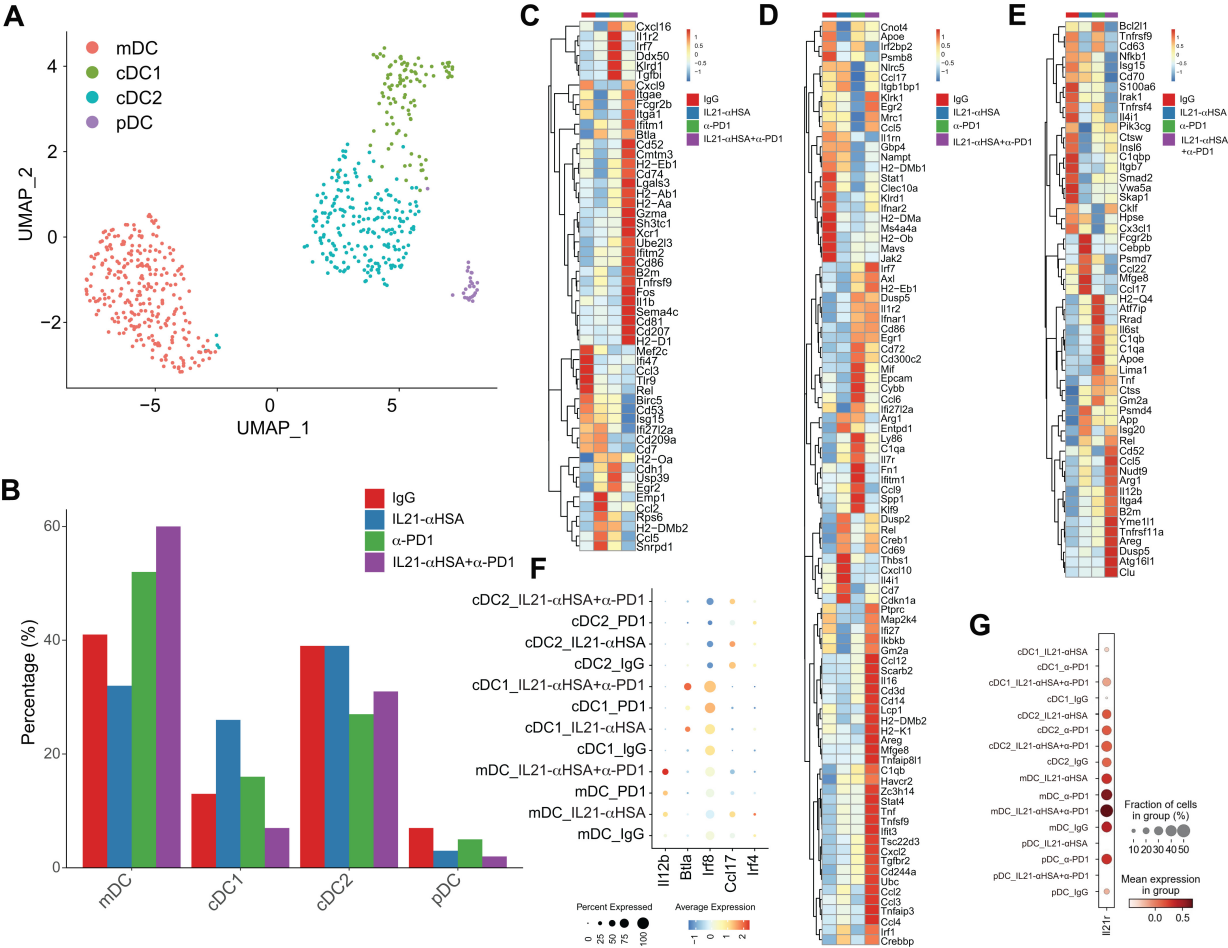
Combination therapy enhances DC activation and maturation. **A,** UMAP showing DC subsets, including mDC, cDC1, cDC2, pDC. **B,** DC subset proportions bar graph. **C**–**E,** Heat map showing the expression of selected DEGs in cDC1, cDC2, mDC cells under different treatment conditions. **F,** Dot plot showing expression of selected genes on different DC subsets under different treatment conditions. **G,** Dot plot showing expression of *Il21r* on different DC subsets under different treatment conditions.

We also analyzed scRNA-seq data to investigate how IL21-anti-HSA and anti-PD-1 mAb regulate DCs in the TME. At the population level combination therapy resulted in a decreased fraction of type 1 dendritic cell (DC1), but an increased fraction of mature/migratory DC (mDC), consistent with the idea that combination therapy promotes differentiation along the DC1 to mDC track (Fig. 6A and B). Analysis of gene profiles in DC1 suggests IL21-anti-HSA and anti-PD-1 mAb synergistically increase interaction and attraction between T cells/NK cells and DCs. *Cxcl16* was increased upon combination treatment. Its receptor Cxcr6 was also found induced to the highest level in both effector and exhausted CD8^+^ T cells (Fig. 4E), consistent with a role of CXCL16/CXCR6 in mediating survival of CD8^+^ T cells by DC (27). In addition, combination therapy increased *Xcr1* in DC1, indicating that the XCL1/XCR1 axis is involved in tighter chemotaxis between CD8^+^/NK cells and DC1. Combination therapy also increased the level of *Cd86*, MHC-I, and MHC-II molecules. It is also notable that the DC1-specific adhesion molecule *Itgae* was also increased by combination treatment (Fig. 6C). These data support the idea that combination therapy increased interaction between T cells and DCs.

Analysis of DEGs in DC2s revealed that the combination IL21-anti-HSA and anti-PD-1 mAb treatment synergistically induced chemokines such as *Ccl3*, *Ccl4*, and *Ccl*5, cytokine receptors such as *Ifnar1* and *Tgfbr2*, costimulatory molecules such as *Tnfsf9* and *Cd86*, inflammatory and fibrosis regulators such as *Il1r2* and *Areg*, transcription factors such as *Irf1*, *Irf7*, *Stat*4, and *Egr2*, and MHC molecules such as *H2-K1*, *H2-Dmb2*, and *H2-Eb1*. These immune molecules are likely involved in increasing interaction between DC2 and other immune cells such as T cells (*Ccl3*, *Ccl4*, *Ccl5*, *Tnfrsf9*, and MHC molecules) and fibroblasts (*Areg*; Fig. 6D). It is noted that, besides positive regulators, immune checkpoint molecule *Havcr2* was also synergistically induced by IL21-anti-HSA and anti-PD-1 mAb combination treatment, indicating a balanced immune response.

Combined IL21-anti-HSA and anti-PD-1 mAb treatment induced synergistic gene upregulation in mDC, including chemokines such as *Ccl5*, antigen presentation molecules such as *B2m*, cytokines such as *Il12b*, inflammatory and fibrosis regulators such as *Areg*, and costimulatory molecules such as *Tnfrsf11a* (Fig. 6E). These changes represent increased communication between mDC with other immune and stromal cell in the TME. In addition, markers that are representative of DC1 origin, such as *Il12b*, *Btla*, and *Irf8*, were upregulated in the combination group, but the markers representing DC2 origin, such as *Irf4* and *Ccl17*, were downregulated, suggesting that DC1 to mDC differentiation was enhanced in the combination treatment group (Fig. 6F). It is notable that *Il21r* expression is induced the highest in mDC compared with DC2 and DC1. In addition, *Il21r* expression increased synergistically upon combination treatment. This data suggests that IL21R signaling might coordinate with T-cell effector molecules such as IFNg to drive DC maturation (Fig. 6G).

### Combination Therapy Synergistically Promoted Monocyte to the Type 1 Macrophage Trajectory in the TME

**FIGURE 7:**
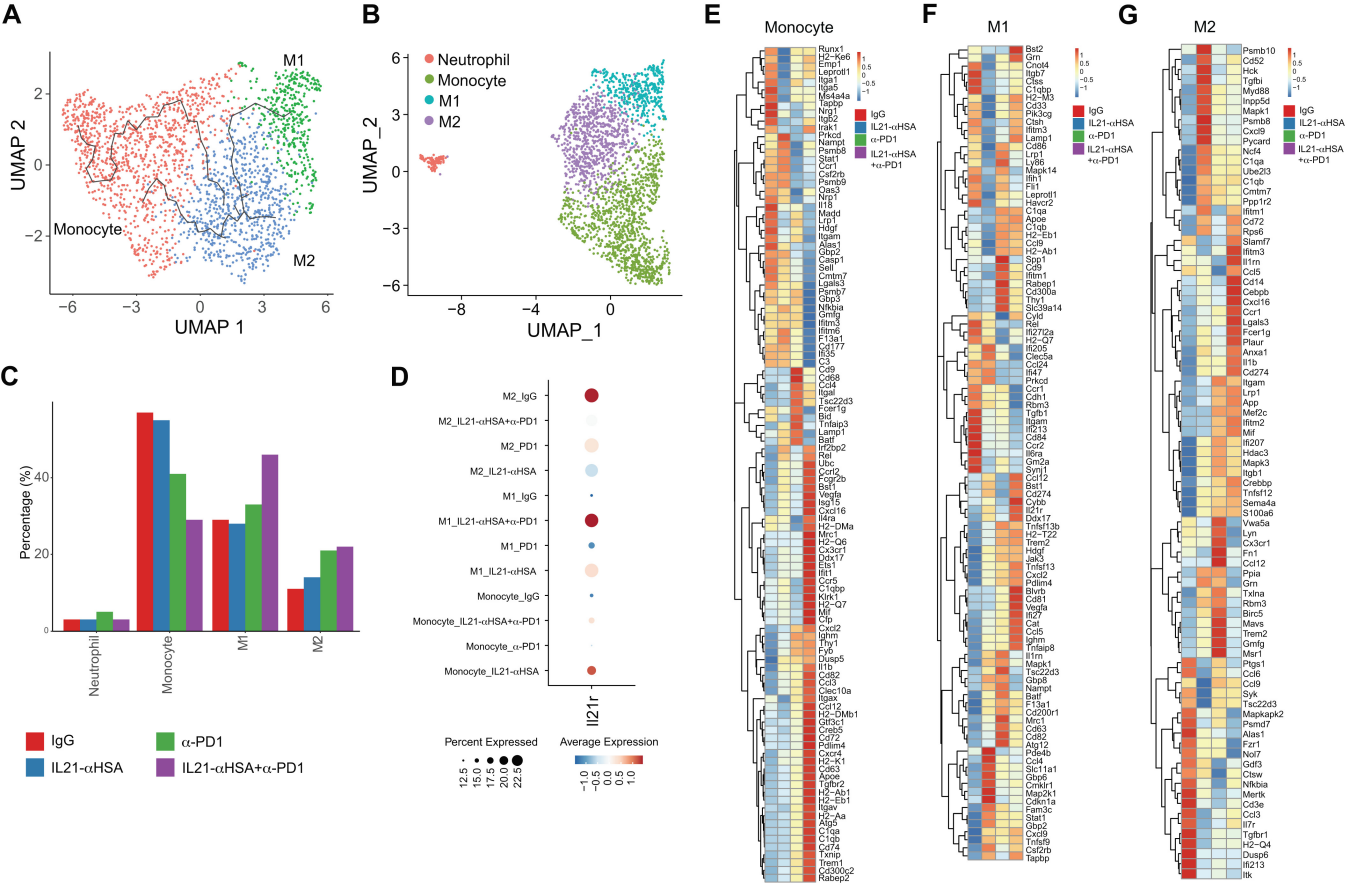
Combination therapy synergistically promoted monocyte to the M1 trajectory in the TME. **A,** Pseudotime plot of macrophage subsets constructed by Monocle 3. **B,** UMAP showing macrophages, neutrophils, and monocytes. **C,** Bar plot of the proportions of the above cell subsets. **D,** Dot plot showing expression levels of *Il21r* on different macrophage subsets under different treatment conditions. **E–G,** Heat map showing the expression of selected DEG in monocytes, TAM1, TAM2 cells under different treatment conditions.

Analysis of scRNA-seq data showed that monocytes differentiated toward type 1 (M1) and the type 2 macrophages (M2) by the cell trajectory analysis (Fig. 7A). Analysis of monocytes and macrophages indicated that therapy resulted in increased proportions of both type I and type II macrophages, although M1 increased at a greater level (Fig. 7B and C). In contrast, the frequency of monocytes was decreased in the combination group, suggesting IL21-anti-HSA/anti-PD-1mAb synergistically promotes differentiation of M1/M2 from monocytes with a more favorable M1 trajectory. IL21-anti-HSA, and IL21-anti-HSA/anti-PD-1 mAb combination induced higher levels of *Il21r* expression in both monocytes and M1, suggesting IL21-anti-HSA plays a direct role in driving M1 differentiation (Fig. 7D). IL21-anti-HSA and anti-PD-1 mAb also additively induced genes that promote angiogenesis such as *Vegfa*, immune activation molecules such as *Cd300c2*, *Trem1*, and *Tnfsf9*, inflammation and fibrosis such as *Il1b*, chemotaxis such as *Cxcr4*, *Cx3cr1*, *Ccr5*, *Cxcl2*, *Ccl12*, and *Cxcl16*, complement activation such as *C1qbp*, *C1qa*, and *C1qb*, and antigen presentation such as *H2-K1*, *H2-Ab1*, *H2-Eb1*, *H2-Aa*, and *Cd74* in monocytes (Fig. 7E). These molecules are likely mediating greater interaction of monocytes with T cells, stromal cells and other immune cells in the TME, and thereby promoting monocyte to macrophage differentiation and monocyte effector function.

DEG analysis of M1 macrophages showed that IL21-anti-HSA and anti-PD-1 mAb combination treatment resulted in synergistic increases of proangiogenic factors such as *Vegfa*, chemokines such as *Ccl12, Cxcl2*, *Ccl5*, and immune-stimulating molecules such as *Trem2*. It is noteworthy that combination treatment also elevated expression of *Tnfsf13b* and *Tnfsf13*, as well as their ligand *Tnfrsf13b*, suggesting that this autocrine loop might be involved in promoting M1 differentiation in the TME (ref. 28; Fig. 7F). DEG analysis of M2 macrophages showed that IL21-anti-HSA and anti-PD-1 mAb combination treatment resulted in synergistic increases in expression of chemokines and chemokine receptors such as *Ccl5*, *Cxcl16,* and *Ccr1*, cytokines such as *Il1b* and *Il1rn*, and transcription factors such as *Cebpb* (Fig. 7G). These data indicate a greater interaction between M2 with other immune cells. In addition, combination treatment also elevated expression of *Tnfsf13b* and *Tnfrsf13b*, suggesting that this autocrine loop is also involved in promoting M2 differentiation in the TME. Besides proimmune factors, combination treatment led to a significant increase of *Cd274* in both M1 and M2, suggesting a self-limiting mechanism of immune regulation and an important role of macrophage-expressed PD-L1 in cancer immunotherapy. Consistent with this notion, *Havcr2* was also increased in M1 upon combination treatment (Fig. 7F).

**FIGURE 8:**
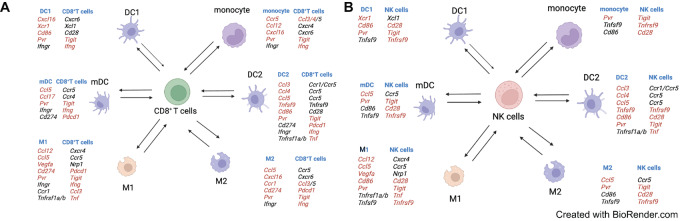
Reshaping cell-cell communication by combination therapy. The ligand and receptor pairs mediate the interaction between myeloid cells and CD8^+^ T cells (**A**) or NK cells (**B**). Genes upregulated by IL21 and anti-PD-1 mAb combination are indicated in red (unchanged genes are in black). The figure is drawn on the BioRender website (Created with BioRender.com).

Given that CD8^+^ T cells and NK cells play a crucial role in facilitating the antitumor effect of combination therapy, we examined the intercellular communication between CD8^+^ T cells, NK cells, and the major myeloid cell populations (Fig. 8). We found that the combination treatment increased communication between CD8^+^ T cells and myeloid cells. Cytokines such as *Ifng*, *Ccl3*, and *Ccl4* are increased in CD8^+^ T cells. In contrast, cytokines and cytokine receptors such as *Cxcl16*, *Ccl3*, *Ccl4*, *Ccl5*, *Ccl12*, *Xcr1*, and *Ccr1* are upregulated in myeloid cells (Fig. 8A). In addition, we observed an upregulation of the costimulatory signaling molecules Cd86/Cd28 and checkpoint inhibitors, including CdD274/Pdcd1 and Pvr/Tigit, enabling stronger communication between CD8^+^ T cells and myeloid cells (Fig. 8A). The analysis also showed increased chemotaxis, costimulation, and checkpoint inhibition between NK cells and myeloid cells (Fig. 8B). These data indicate that IL21 and anti-PD-1 combination treatment leads to the transformation of cell-cell communication that underpins the coordinated antitumor immune responses.

## Discussion

In this study, we have demonstrated that IL21 is mainly produced by two CD4^+^ T-cell subsets, namely T_fh_ cells and hyperactivated/exhausted CXCL13^+^CD4^+^ T cells. In a mouse MC38 tumor model, we demonstrated that IL21 is produced by hyperactivated/exhausted CD4^+^ T cells in the TME. In addition, we found that anti-PD-1 mAb treatment in both human patients and the mouse tumor model induced IL21 expression as well as greater accessibility of the *Il21* gene locus. Moreover, we showed that IL21 is important for mediating the antitumor function of anti-PD-1 mAb. Furthermore, we showed that systemically administered IL21 exerts its antitumor function mainly on the immune cells in the TME. scRNA-seq analysis of tumor-associated immune cells revealed that IL21-anti-HSA and anti-PD-1 synergistically promote the effector differentiation of T cells and DCs, and the type 1 polarization of monocytes and macrophages.

We showed that IL21 is produced by T_fh_ cells in the human TME, particularly in colon cancer. Several recent studies demonstrate that IL21 can be produced by tumor-associated T_fh_ cells and IL21 promotes antitumor immunity through activating CD8^+^ T cells (12, 29). However, it is important to note that T_fh_ cells are almost absent in the TME of many syngeneic mouse tumor models, including the commonly used MC38 model, which we observed in this study. The role of T_fh_-derived IL21 in ICI cancer immunotherapy needs to be established using a relevant mouse model. Our analysis also showed that IL21 is produced by human CXCL13^+^CD4^+^ T cells and its murine equivalent in the TME. In addition, IL21 was further induced in CD4^+^ T cells upon PD-1 mAb treatment in the MC38 model. Using a murine model, we further demonstrated that IL21 mediates some antitumor effects of anti-PD-1 mAb. Several recent studies demonstrate that human CXCL13^+^CD4^+^ and CXCL13^+^CD8^+^ T cells, which are also phenotypically similar to exhausted or hyperactivated T cells, are tumor antigen–specific and mediate responses to the anti-PD-1 therapy (9, 30–34). These data suggest that IL21 can be induced after CD4^+^ T cells engage tumor antigens and mediate the helper function of CD4^+^ T cells, that is important for CD8^+^ T cells.

Consistent with the function of endogenous IL21, we showed that administration of IL21 promotes CD8^+^ T cell–mediated immune responses in the TME. Specifically, our data indicate that IL21 promotes the differentiation toward a hyperactivated/exhausted phenotype, and PD-1 mAbs had a similar effect. Combination of IL21 and anti-PD-1 results in greater frequencies of hyperactivated/exhausted CD8^+^ T cells in the TME. Combination therapy also synergistically increased CD8^+^ T-cell clonal expansion in the hyperactivated/exhausted functional state. Because it has been shown that the hyperactivated/exhausted CD8^+^ T-cell subset is enriched with tumor antigen–specific T cells, our data suggest that IL21 and anti-PD-1 in combination enhance the clonal expansion of the tumor antigen–specific CD8^+^ T cells.

Two recent studies used the anti-PD-1-IL21 fusion proteins for targeting IL21 to the T cells in the TME. This design, in principle, will allow the focus of IL21 on tumoral T cells while reducing systemic toxicity (23, 35). Li and colleagues developed a design incorporating an anti-PD-1 single-chain variable fragment and wild-type IL21. They showed this fusion protein had superior antitumor efficacy than PD-1 mAbs plus IL21 *in vivo*. Nevertheless, these authors did not provide the pharmacokinetic data of the fusion protein and the recombinant IL21. Recombinant IL21 has a short half-life (*t*1/2 = 2.6 hours) *in vivo* (36). Thus, it is unclear whether the difference is due to the level of these proteins *in vivo*. It is also not clear whether the toxicity profile improves. Shen and colleagues further advanced the concept by attenuating the activity of IL21 to improve the pharmacokinetic property of the anti-PD-1-IL21mutein fusion protein. Nevertheless, the *in vivo* efficacy is underwhelming. In addition, this study did not compare the antitumor efficacy between the anti-PD-1-IL21mutein fusion protein and the combination of PD-1 mAbs and IL21 (23). Regarding mechanisms of action, Li and colleagues showed that PD-1-IL21 promotes stem T-cell generation *in vitro*, consistent with a previous study that shows similar findings using a recombinant IL21 (36). In contrast, our analysis is focused on TILs upon treatment *in vivo*. Whether IL21 promotes the generation of memory stem T cells *in vivo* remains to be substantiated. Overall, it remains to be established whether PD-1-IL21 fusion strategy is superior to IL21 plus anti-PD1 in the preclinical setting. Furthermore, our approach offers clinical flexibility by enabling the combination of IL21’s antitumor properties with various immunotherapies.

Previous studies have shown that IL21 has a certain inhibitory effect on DCs *in vitro* (37, 38). The addition of IL21 to mouse bone marrow–derived DCs reduces the expression of MHCIIs (class II MHC molecules), and thereby diminishing their abilities to induce antigen-specific CD4^+^ T-cell proliferation (37). IL21 induces conventional splenic DCs (cDC) apoptosis via STAT3 and Bim (38). In contrast, our data indicate that IL21 promotes DC maturation and activation in the TME. Therefore, IL21 might have diverse functions in different DC subsets.

Besides cell–cell interaction, as illustrated in the case of CD4^+^ T cell help for CD8^+^ T cells through IL21 and IL21R interaction, analysis of the scRNA-seq data reveals many cellular interactions and potential cellular subnetworks. For instance, DCs upregulated *Xcr1*, *Cxcl16*, *Cd86,* and MHC molecules, which allows strong interaction with CD8^+^ T cells that express *Xcl1*, *Cxcr6*, and TCR. These interactions are reflected in myeloid cells as upregulated IFN-induced signature genes. Therefore, IL21 and anti-PD-1 result in the reorganization of cellular networks in the TME that favors tumor elimination.

Our results also showed that, besides immune-stimulating factors, immune inhibitory factors are induced by IL21-anti-HSA and anti-PD-1 combination therapy. These findings keep with the notion that immune responses are self-limiting and provide rich sources for targets for further combination therapy. T cell–derived factors such as CTLA4, LAG3, HAVCR2, and CD39 will be obvious targets for further combination therapy. It is well documented that myeloid cells can also inhibit tumor immunity through multiple mechanisms. Our data demonstrate that PD-L1 can be an important molecule, consistent with the literature. Besides PD-L1, several other factors derived from myeloid cells, including VEGFA, HAVCR2, TNFRSF13B, and BTLA, have the potential to be investigated as possible combinations with IL21 and anti-PD-1 therapy.

## Supplementary Material

Figure S1 Supplementary Figure 1. Distribution of CD4+ T cell subsets.

Figure S2 Supplementary Figure 2. FTY720 injection did not affect on anti-PD-1 anti-tumor effect.

Figure S3 Supplementary Figure 3. IL21-anti-HSA/anti-PD-1 leads to altered immune cell populations in the tumor microenvironment.

Figure S4 Supplementary Figure 4. IL21-anti-HSA and PD-1 mAbs synergy at the proliferation is due to upregulation of IL21R by PD-1 mAbs treatment and IL21 directly promotes proliferation on IL21R+ CD8+ T cells.
