# Supplementary material for: Synergism Between IL21 and Anti-PD-1 Combination Therapy is Underpinned by the Coordinated Reprogramming of the Immune Cellular Network in the Tumor Microenvironment
Source: Cancer Res Commun. 2023 Aug 4;3(8):1460–72. doi: 10.1158/2767-9764.CRC-23-0012 (PMC10402650; doi:10.1158/2767-9764.CRC-23-0012)
Supplement: Figure S1 — Supplementary Figure 1. Distribution of CD4+ T cell subsets. [file crc-23-0012-s01.pdf]

Supplementary Figure S1

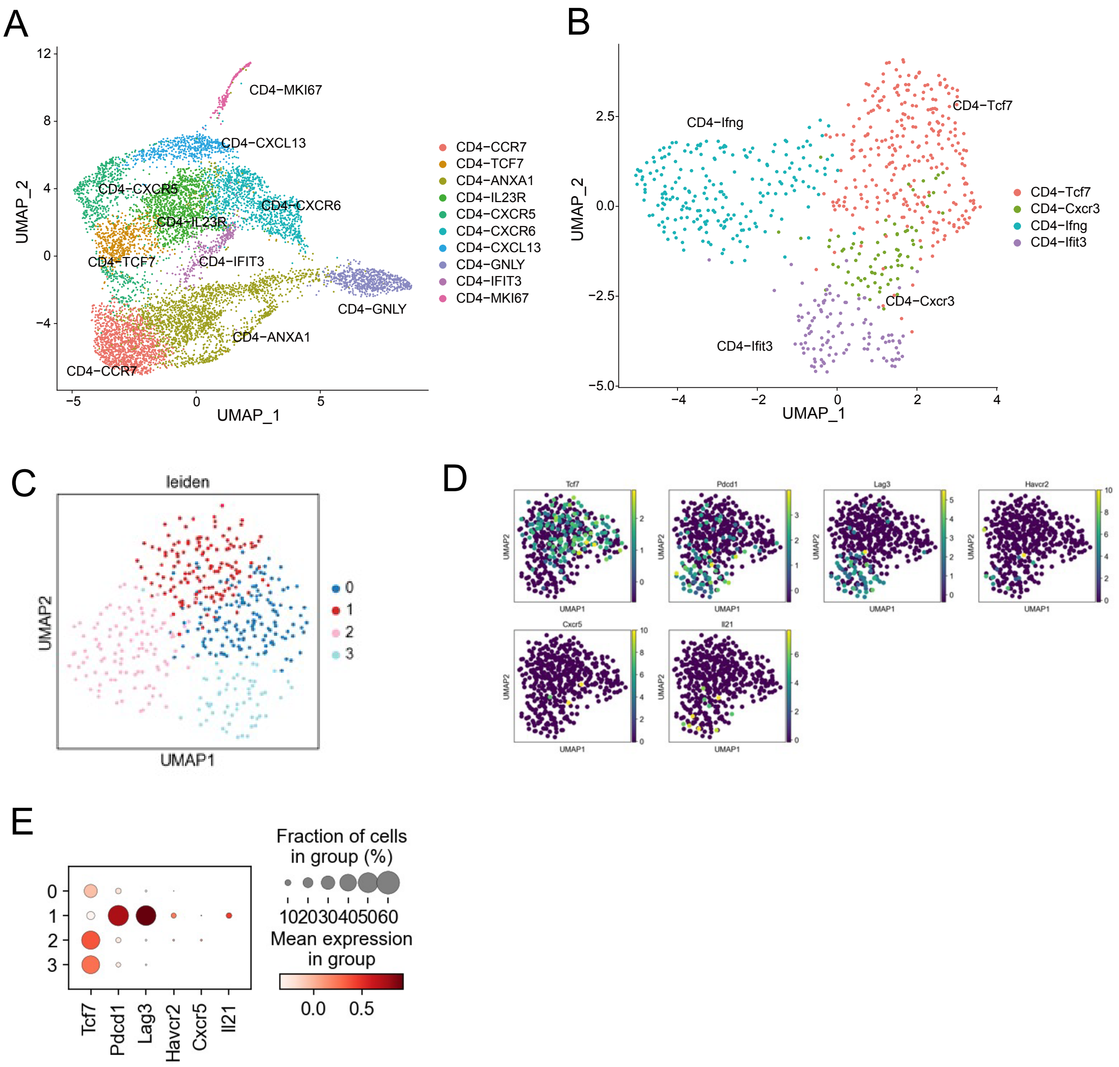

Supplementary Figure. S1 Distribution of CD4+ T cell subsets.
A. UMAP showing CD4+ T cell subsets in human NSCLC, HCC, and colon cancer. B. UMAP showing CD4+ T cell subsets in mouse MC38 tumors. C. UMAP showing CD4 T cell clusters in MC38 tumors. D. UMAP showing expression of selected genes in CD4+ T cell subsets. E. Dot plot showing expression of selected genes on different CD4+ T cell subsets.
