# Supplementary material for: Synergism Between IL21 and Anti-PD-1 Combination Therapy is Underpinned by the Coordinated Reprogramming of the Immune Cellular Network in the Tumor Microenvironment
Source: Cancer Res Commun. 2023 Aug 4;3(8):1460–72. doi: 10.1158/2767-9764.CRC-23-0012 (PMC10402650; doi:10.1158/2767-9764.CRC-23-0012)
Supplement: Figure S2 — Supplementary Figure 2. FTY720 injection did not affect on anti-PD-1 anti-tumor effect. [file crc-23-0012-s02.pdf]

Supplementary Figure S2

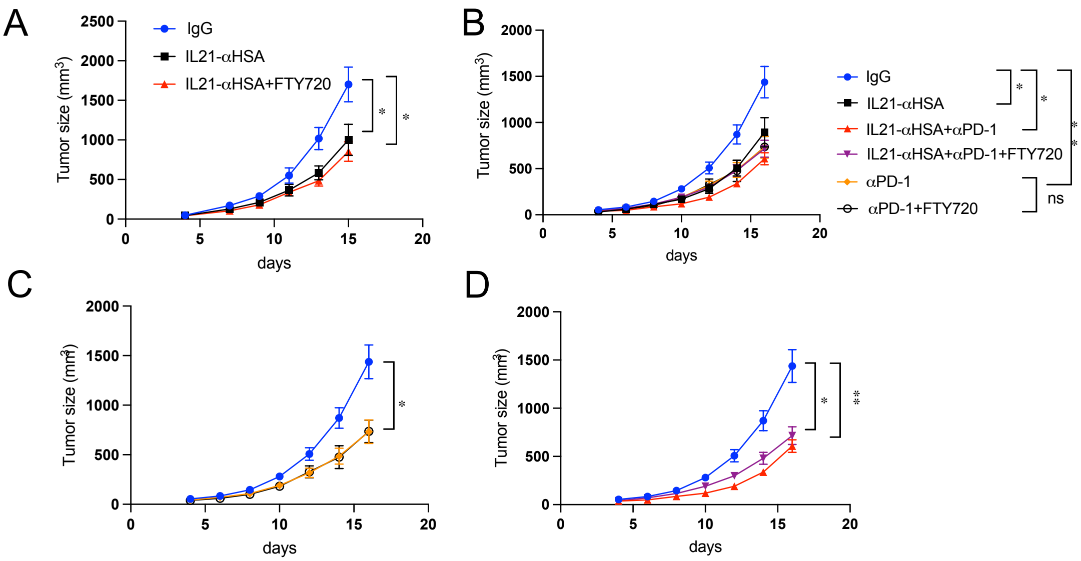

Supplementary Figure. S2 FTY720 injection did not affect on anti-PD-1 anti-tumor effect.
A-D. C57BL/6 mice were inoculated with MC38 colon adenocarcinoma cells subcutaneously, and 5 days later, mice were injected i.p. with FTY720 every other day. IL21-anti-HSA and PD-1 mAb were given every 4 days for a total of 4 treatments. Mouse tumor growth curves (n=4-5) were drawn. Data are presented as Mean±SEM, and two-way ANOVA test was used to compare statistical differences in tumor growth curves between different groups. *P<0.05, **P<0.01, ***P<0.001, ****P<0.0001.
