# Supplementary material for: Synergism Between IL21 and Anti-PD-1 Combination Therapy is Underpinned by the Coordinated Reprogramming of the Immune Cellular Network in the Tumor Microenvironment
Source: Cancer Res Commun. 2023 Aug 4;3(8):1460–72. doi: 10.1158/2767-9764.CRC-23-0012 (PMC10402650; doi:10.1158/2767-9764.CRC-23-0012)
Supplement: Figure S3 — Supplementary Figure 3. IL21-anti-HSA/anti-PD-1 leads to altered immune cell populations in the tumor microenvironment. [file crc-23-0012-s03.pdf]

Supplementary Figure S3

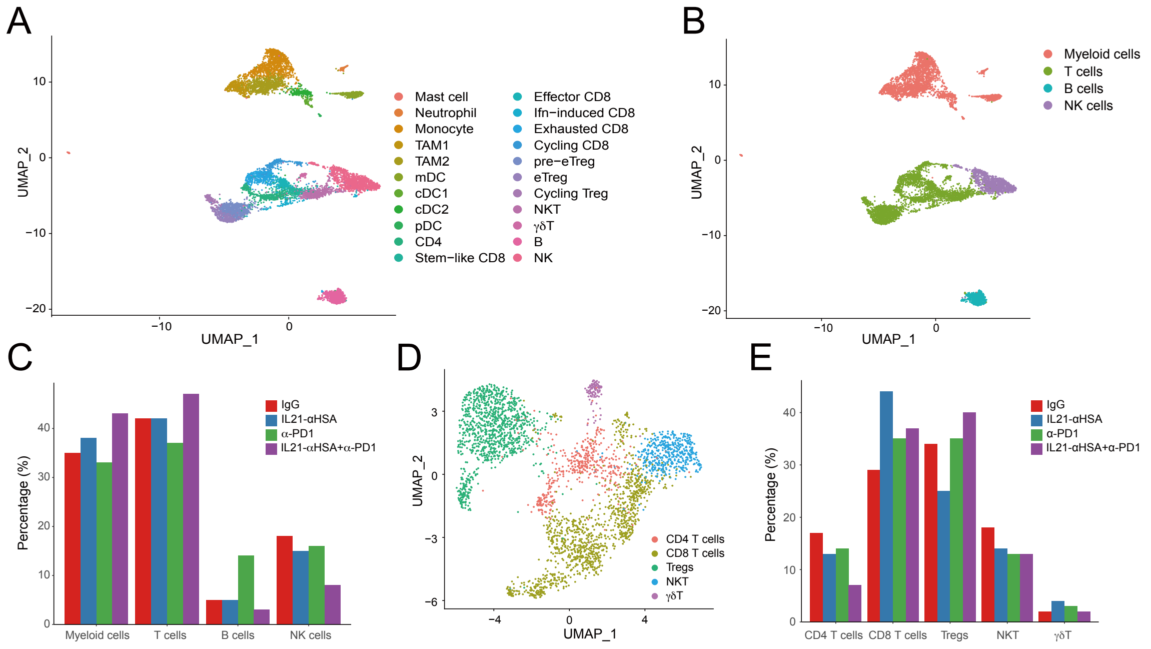

Supplementary Figure. S3 IL21-anti-HSA/anti-PD-1 leads to altered immune cell populations in the tumor microenvironment.

A. UMAP showing macrophages, neutrophils, and monocytes, dendritic cell and T cell subsets. B. UMAP showing myeloid, T cells, B cells and NK cells. C. Bar plot of the distribution of myeloid, T cells, B cells and NK cells in different treatment groups. D. UMAP showing CD4+ T cells, CD8+ T cells, NKT cells, γδT cells. E. Bar graph of the distribution of CD4+ T cells, CD8+ T cells, NKT cells, γδT cells in different treatment groups.
