# Supplementary material for: Synergism Between IL21 and Anti-PD-1 Combination Therapy is Underpinned by the Coordinated Reprogramming of the Immune Cellular Network in the Tumor Microenvironment
Source: Cancer Res Commun. 2023 Aug 4;3(8):1460–72. doi: 10.1158/2767-9764.CRC-23-0012 (PMC10402650; doi:10.1158/2767-9764.CRC-23-0012)
Supplement: Figure S4 — Supplementary Figure 4. IL21-anti-HSA and PD-1 mAbs synergy at the proliferation is due to upregulation of IL21R by PD-1 mAbs treatment and IL21 directly promotes proliferation on IL21R+ CD8+ T cells. [file crc-23-0012-s04.pdf]

Supplementary Figure S4

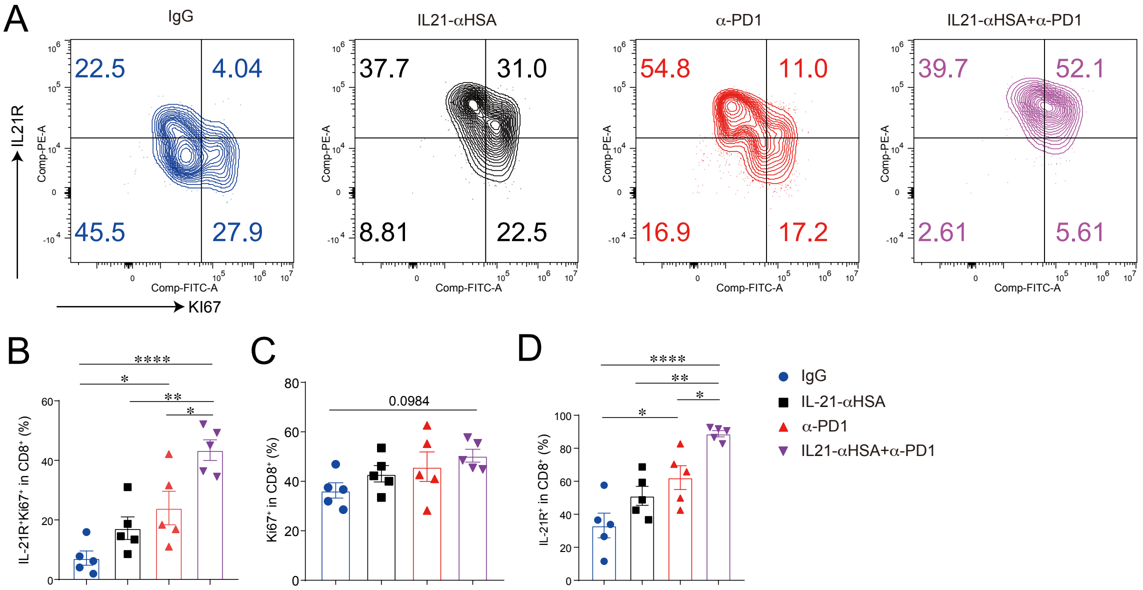

Supplementary Figure. S4 IL21-anti-HSA and PD-1 mAbs synergy at the proliferation is due to upregulation of IL21R by PD-1 mAbs treatment and IL21 directly promotes proliferation on IL21R+ CD8+ T cells.

A-D. Subcutaneous injection of MC38 tumor cell suspensions on day 0, IgG, IL21-anti-HSA, PD-1 mAb, IL21-anti-HSA/PD-1 mAb treatment on day 9, and flow cytometry analysis after 96h. A-D. Representative flow cytometry contour plots and quantitative statistics of IL21R and Ki67 expression on CD8+ T cells under different treatment conditions. Data are presented as Mean±SEM, and one-way ANOVA test was used to compare statistical differences between different groups. *P<0.05, **P<0.01, ***P<0.001, ****P<0.0001.
